# Sarcopenia is a prognostic factor for overall survival in elderly patients with head-and-neck cancer

**DOI:** 10.1007/s00405-019-05361-4

**Published:** 2019-03-04

**Authors:** N. Chargi, S. I. Bril, M. H. Emmelot-Vonk, R. de Bree

**Affiliations:** 1 Department of Head and Neck Surgical Oncology, UMC Utrecht Cancer Center, University Medical Center Utrecht, Heidelberglaan 100, 3584 CX Utrecht, The Netherlands; 2 Department of Medical Oncology, UMC Utrecht Cancer Center, University Medical Center Utrecht, Heidelberglaan 100, 3584 CX Utrecht, The Netherlands; 3 Department of Geriatrics, University Medical Center Utrecht, Heidelberglaan 100, 3584 CX Utrecht, The Netherlands; 4 Department of Head and Neck Surgical Oncology, University Medical Center Utrecht, House Postal Number Q.05.4.300, PO BOX 85500, 3508 GA Utrecht, The Netherlands

**Keywords:** Body composition, Muscle function, Head-and-neck neoplasms, Survival, Sarcopenia

## Abstract

**Objectives:**

Sarcopenia is known as a geriatric syndrome associated with increased disability and decreased survival in elderly patients. In oncological patients, pretreatment low skeletal muscle mass (SMM), sometimes referred to as sarcopenia, is an emerging negative prognostic factor. Commonly, only SMM is assessed in cancer patients. Sarcopenia is defined as the combination of low SMM and low muscle function (MF). We investigated the relation between SMM, MF, sarcopenia (SMM and MF combined), and overall survival (OS) in a group of elderly patients with head-and-neck squamous cell carcinoma (HNSCC).

**Patients and methods:**

A retrospective study in elderly HNSCC patients treated between 2015 and 2018 was performed. The prognostic value of SMM and MF seperately, and sarcopenia was investigated.

**Results:**

Eighty-five patients were included of whom 48.2% had sarcopenia. The median OS was significantly worse for patients treated with curative intent with sarcopenia (12.07 months; IQR 3.64–21.82) compared to patients without sarcopenia (13.60 months; IQR 5.98-27.00) (HR 2.80; 95% CI 1.14–6.88; *p* = 0.03). SMM and MF seperately were not significant predictors of OS.

**Conclusion:**

Sarcopenia is associated with impaired OS in elderly HNSCC patients. Sarcopenia, defined as the combination of low SMM and low MF, appears to be a better predictor of OS than low SMM or low MF separately.

## Introduction

Research into the field of body composition and specifically low skeletal muscle mass (SMM), sometimes also referred to as sarcopenia, has increasingly gained interest over the last decade in the field of oncology. In geriatrics, sarcopenia is known as an age-related syndrome with a multifactorial etiology, characterized by generalized loss of SMM and loss of muscle strength [1].

Risk factors for the presence of sarcopenia are malnutrition, immobilization, and illness. Sarcopenia is a risk factor for various adverse outcomes including physical disability, decreased quality of life, and ultimately death [1]. In human aging, muscle wasting is an imminent process. It is estimated that the prevalence of sarcopenia in the general population is 5–13% for people aged 60–70 years, and up to 50% for those aged 80 years or above [2]. Independent of age, sarcopenia is impaired in various diseases due to inflammation, malnutrition, and immobilization. Cachexia is a complex metabolic syndrome in which inflammation is the key feature and weight loss (≥ 5% of body weight during the past 12 months) is the key diagnostic criterium. Cachexia can be an underlying condition in patients with sarcopenia [3].

The majority of research within the oncological community has defined sarcopenia as radiologically assessed low SMM and/or low skeletal muscle quality. Previous research in elderly people showed that the correlation between SMM and muscle strength is moderate-to-weak, and the relationship between muscle strength and SMM is not linear [4, 5]. For this reason, the European working group on sarcopenia in older people (EWGSOP) recommended diagnosing sarcopenia based on the presence of both low SMM and low muscle function (MF; strength or performance) [1].

Within the field of oncology, radiologically assessed low SMM appears to be a negative predictive and prognostic factor for various outcomes including disease progression and survival in a variety of cancer types [6]. For example, radiologically assessed low SMM is associated with chemotherapy dose-limiting toxicity in patients with head-and-neck cancer [7], breast cancer [8], and renal cell carcinoma [9]; increased incidence of postoperative complications in patients with head-and-neck cancer [10, 11], esophageal squamous cell carcinoma [12] and colorectal cancer [13]; and decreased survival in patients with head-and-neck cancer [11, 14], colorectal cancer [15], and pancreatic adenocarcinoma [16].

In the majority of studies on the effect of sarcopenia on survival of cancer patients, and in all studies regarding head-and-neck cancer patients, only radiologically assessed low SMM was used to define sarcopenia. There are very few studies available in cancer patients that assess the prognostic value of sarcopenia as defined by the combination of low SMM and low MF. One study performed with gastric cancer patients who underwent gastrectomy showed that patients with sarcopenia, as defined by the combination of low SMM and low MF, showed a significantly higher complication rate compared to patients without sarcopenia [17]. In head-and-neck cancer, no studies are available on the relationship between sarcopenia, as defined by the combination of low SMM and low MF, and adverse outcomes. The aim of this study is to explore the relationship between sarcopenia and overall survival in elderly patients with head-and-neck cancer.

## Materials and methods

### Patients and study design

This study was designed as a single-center retrospective study. We reviewed elderly patients (≥ 70-years old) with pathologically proven head-and-neck squamous cell carcinoma (HNSCC) who had a geriatric assessment during their diagnostic workup between April 2015 and February 2018. In our center, elderly HNSCC patients are offered geriatric assessment, but patients may refuse. Histologic tumor types other than squamous cell carcinoma were excluded. The design of this retrospective study was approved by the Medical Ethical Research Committee of our center (approval ID 17-365/C).

Factors with known or suspected relation with HNSCC treatment outcomes and with sarcopenia were collected: age, sex, body mass index (BMI), weight loss in the past 6 months, risk of malnutrition assessed with the malnutrition universal screening tool (MUST), smoking status, alcohol use, comorbidity expressed as a Charlson Comorbidity Index (CCI) score, tumor type (primary, second primary or recurrence), tumor site, human papillomavirus (HPV) status (for oropharyngeal cancer), tumor-node-metastasis (TNM) stage, hematological and biochemical markers at diagnosis, including hemoglobin (Hb), leukocytes, C-reactive protein (CRP), creatinine and albumin, and treatment intention.

### Definition of sarcopenia

Sarcopenia was defined as the combination of low SMM and low MF, as determined by muscle strength or physical performance measurements [1].

### Skeletal muscle mass

Skeletal muscle mass was measured as cross-sectional muscle area (CSMA) on pretreatment CT or MRI imaging of the head-and-neck area at the level of the third cervical vertebrae (C3). The axial slide of the imaging which showed both transverse processes and the entire vertebral arc was selected for the segmentation of muscle tissue. For CT imaging, muscle area was defined as the pixel area between the radiodensity range of − 29 and + 150 Hounsfield units (HU), which is specific for muscle tissue [18]. For MRI, muscle area was manually segmented, and fatty tissue was manually excluded.

Segmentation of muscle tissue was manually performed using the commercially available software package SliceOmatic (Tomovision, Canada). Cross-sectional muscle area at the level of C3 was converted to CSMA at the level of L3 using a previously published formula [19]. The lumbar skeletal muscle index (SMI) was calculated by correcting SMM at the level of L3 for height. Patients had a low SMI if this value was below 43.2 cm^2^/m^2^; this cut-off value was established in a separate cohort of head-and-neck cancer patients [7].

### Muscle strength

Isometric handgrip strength (HGS) is strongly related with overall muscle strength [20]. Handgrip strength was measured using a Jamar hydraulic handheld dynamometer according to the recommendations of the American society of hand therapist’s (ASHT) and expressed in kilograms (kg). Patients were asked to squeeze maximally with each hand. The average score of the left and right hands was used for analysis. Patients had low HGS if the HGS was below 30 kg (men) or below 20 kg (women) [1].

### Muscle performance

The 4-m gait speed is a reflection of individual’s lower limb muscle function. It is a widely accepted way to assess muscle performance [21]. Gait speed was measured as the average speed during a 4-m walking test. The time measured to complete a 4-m walk was measured. Patients had low muscle performance if the 4-m gait speed was below 0.8 m/s [1].

### Statistical analysis

Data analyses were performed using IBM SPSS statistics 25. Descriptive statistics for continuous variables with a normal distribution were presented as mean with standard deviation (SD). Variables with a skewed distribution were presented as median with interquartile range (IQR). Categorical variables were presented as frequencies and percentages. Likelihood ratio (LR) Chi-square statistics were used for analyzing associations of the percentages of each categorical variable with the presence or absence of sarcopenia. Independent sample *t* tests were used for comparing the means of the hematological and biochemical markers with the presence or absence of sarcopenia. Pearson’s correlation was used to assess the correlation between SMM, MF parameters, age, and BMI. Only patients with curative treatment intent were selected for overall survival analysis. Survival was visualized using Kaplan–Meier survival curves and number at risk tables. We defined overall survival as the time elapsed between the date of histologic diagnosis and death or date of last follow-up, whichever occurred first. We calculated the 3-year overall survival rate for patients with sarcopenia and without sarcopenia, Wilcoxon test was used for analyzing the statistical significance of the difference in 3-year overall survival rate. A cox proportional hazard regression model was used for univariate and multivariate analyses of survival. Covariates used in the multivariate analysis were selected based on clinical significance or selected based on statistical significance (*p* < 0.05) in univariate cox regression analysis. Statistical significance was evaluated at the 0.05 level using two-sided tests.

## Results

### Patient characteristics

Descriptive data are described in Table 1. A total of 85 patients were included with a mean age of 81.5 years (SD 6.5). The majority of patients were female (55.3%) with a mean BMI of 26.9 kg/m^2^ (SD 4.8). Most patients were former smokers (54.1%) with mean pack-years of 21–40 years. Most patients had multiple comorbidities, as represented by a high Charlson Comorbidity Index score (CCI). Most patients underwent treatment with curative intent (83.5%). The median follow-up time was 11.14 months (IQR 3.64–21.83 months); 33 patients (38.8%) died during the study period.

**Table 1:** Patient characteristics

Characteristics	Mean (SD)	Frequencies *n*, (%)
Sex		
Female		47 (55.3)
Male		38 (44.7)
Age (years)	81.5 (6.5)	
BMI (kg/m^2^)	26.9 (4.8)	
Smoking status		
Never		30 (35.3)
Former		46 (54.1)
Current		9 (10.6)
Pack-years		
1–20		8 (9.4)
21–40		10 (11.8)
41–60		4 (4.7)
≥ 61		7 (8.2)
Alcohol use		
Never		28 (32.9)
Former		8 (9.4)
Current		49 (57.6)
Alcohol intake (units/day)		
< 2		37 (43.5)
2–4		12 (14.1)
≥ 5		–
Charlson comorbidity index		
Mild (0–3)		4 (4.7)
Moderate (4–5)		10 (11.8)
Severe (≥ 6)		71 (83.5)
Weight loss in the past 6 months		
None		56 (65.9)
< 10%		23 (27.1)
≥ 10%		6 (7.1)
MUST score		
< 2		66 (77.6)
≥ 2		19 (22.4)
TNM stage		
I		11 (12.9)
II		19 (22.4)
III		16 (18.8)
IV		39 (45.9)
Tumor type		
Primary		65 (76.5)
Second primary		6 (7.1)
Recurrent		14 (16.5)
Tumor site		
Oral cavity		52 (61.2)
Nasopharynx		2 (2.4)
Oropharynx*		5 (5.9)
Hypopharynx		3 (3.5)
Larynx		8 (9.4)
Skin		12 (14.1)
Salivary glands		1 (1.2)
Paranasal sinuses		2 (2.4)
Treatment intention		
Curative		71 (83.5)
Palliative		14 (16.5)

*Four patients had HPV-negative oropharyngeal cancer; one patient had missing data on HPV status

Of the 85 included patients; 69 patients (81.2%) had low SMI, 50 patients (58.8%) had low HGS, and 58 patients (68.2%) had low gait speed. Forty-one patients (48.2%) were classified as sarcopenic; of these patients, 31 patients (75.6%) had low SMI in combination with low HGS and low gait speed, six patients (14.6%) had low SMI in combination with low gait speed and normal HGS, and four patients (9.8%) had low SMI in combination with low HGS and normal gait speed.

Tables 2 and 3 show the general characteristics and the hematological and biochemical markers of the included patients according to the presence or absence of sarcopenia. Patients with sarcopenia were most likely to smoke (77.8% versus 22.2%; LR 8.37, *p* = 0.02), to have lower mean hemoglobin levels at diagnosis [8.09 mmol/L (SD 1.06) versus 8.67 mmol/L (SD 1.12); *p* = 0.03] and to die (63.6% versus 36.4%; LR 5.17, *p* < 0.01).

**Table 2:** General characteristics of the study patients by the presence of sarcopenia

	Sarcopenia		Without sarcopenia		Likelihood ratio (LR)	*p* value
	*n*	(%)	*n*	(%)		
Age (years)					8.82	0.08
70–75	7	43.8	9	56.3		
76–80	8	32	17	68		
81–85	12	48	13	52		
86–90	5	62.5	3	37.5		
> 90	9	81.8	2	18.2		
BMI (kg/m^2^)					7.70	0.07
≤ 18.5	3	100	–	–		
18.5–25	17	56.7	13	43.3		
25–30	14	46.7	16	53.5		
≥ 30	7	31.8	15	68.2		
MUST score					0.19	0.80
< 2	31	47.0	35	53.0		
≥ 2	10	52.6	9	47.4		
Smoker					8.37	0.02*
No	18	60	12	40		
Yes	7	77.8	2	22.2		
Former	16	34.8	30	65.3		
Pack-years					2.26	0.55
1–20	5	62.5	3	37.5		
21–40	3	30	7	70		
41–60	2	50	2	50		
≥ 61	4	57.1	3	42.9		
Alcohol use					4.57	0.23
No	17	60.7	11	39.3		
Former	4	50	4	50		
Current						
≤ 2 units/day	17	45.9	20	54.1		
≥ 2 units/day	3	25	9	75		
CCI					4.00	0.07
≤ 6	11	34.3	21	65.6		
> 6	30	56.6	23	43.4		
TNM stage					0.94	0.84
I	5	45.4	6	54.5		
II	8	42.1	11	57.9		
III	7	43.8	9	56.3		
IV	21	53.8	18	46.2		
Treatment intention					1.74	0.25
Curative	32	45.1	39	54.9		
Palliative	9	64.3	5	35.7		
Radiotherapy					0.45	0.87
No	24	49	25	51		
Yes, primary	8	42.1	11	57.9		
Yes, adjuvant	9	52.9	8	47.1		
Chemotherapy (not applicable)	–		–		–	–
Surgery					0.47	0.62
No	12	54.5	10	45.5		
Yes	29	46	34	54		
Synchronous tumor					1.82	0.36
No	40	50	40	50		
Yes	1	20	4	80		
Metachronous tumor					0.95	0.62
No	40	49.4	41	50.6		
Yes	1	25	3	75		
Recurrence					0.20	0.65
No	35	47.3	39	52.7		
Yes	6	54.5	5	45.5		
Dead					5.17	0.03*
No	20	38.5	32	61.5		
Yes	21	63.6	12	36.4		
SMI					24.54	< 0.01**
Low	41	59.4	28	40.6		
High	–		16	100		
HGS					24.57	< 0.01**
Low	35	70	15	30		
High	6	17.1	29	82.9		
Gait speed					19.14	< 0.01**
Low	37	63.8	21	36.2		
High	4	14.8	23	85.2		

*Correlation is significant at the 0.05 level (two-tailed)

**Correlation is significant at the 0.01 level (two-tailed)

**Table 3:** Hematological and biochemical markers of the study patients by the presence or absence of sarcopenia

	Sarcopenia (mean, SD)	Without sarcopenia (mean, SD)	Mean difference (SD)	95% CI	*p* value
Hb (mmol/L)	8.09 (1.06)	8.67 (1.12)	− 0.58 (0.26)	− 1.10 to − 0.05	0.03*
CRP (mg/L)	9.93 (15.10)	8.12 (11.86)	1.81(3.38)	− 4.93 to 8.56	0.59
Leukocytes (× 10^9/L)	10.78 (8.24)	8.15 (2.45)	2.63(1.44)	− 0.25 to 5.51	0.07
Albumin (g/L)	39.56 (2.28)	40.98 (2.53)	− 1.42 (1.14)	− 3.83 to 0.99	0.23
Creatinine (µmol/L)	87.55 (30.95)	95.38 (51.06)	− 7.84 (10.43)	− 28.65 to 12.98	0.46

*Correlation is significant at the 0.05 level (2-tailed)

### Correlation analysis

Results from the correlation analyses are shown in Table 4. Significant low-to-moderately strong correlation coefficients are seen for SMI and BMI (*r* = 0.49), SMI and age (*r* = − 0.37), HGS and age (*r* = − 0.46), gait speed and age (*r* = 0.28), and for gait speed and HGS (*r* = − 0.39).

**Table 4:** Pearson correlation analysis for variables associated with sarcopenia

Measures	SMI	HGS	Gait speed	Age	BMI
SMI	–	0.16	− 0.15	− 0.37*	0.49*
HGS	0.16	–	− 0.39*	− 0.46*	− 0.04
Gait speed	− 0.15	− 0.39*	–	0.28*	0.05
Age	− 0.37*	− 0.46*	0.28**	–	− 0.02
BMI	0.49*	− 0.04	0.05	− 0.02	–

*Correlation is significant at the 0.01 level (two-tailed)

**Correlation is significant at the 0.05 level (two-tailed)

### Survival analysis

**Fig. 1:**
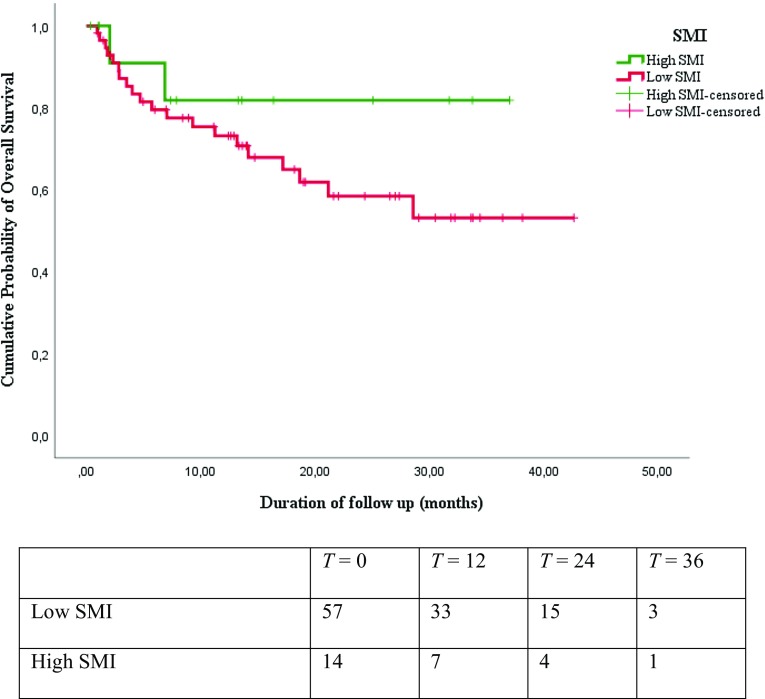
Kaplan–Meier overall survival curves and number at risk table for patients with low SMI and high SMI showed no statistically significant difference (Log-rank chi-square 1.14; *p* = 0.29)

**Fig. 2:**
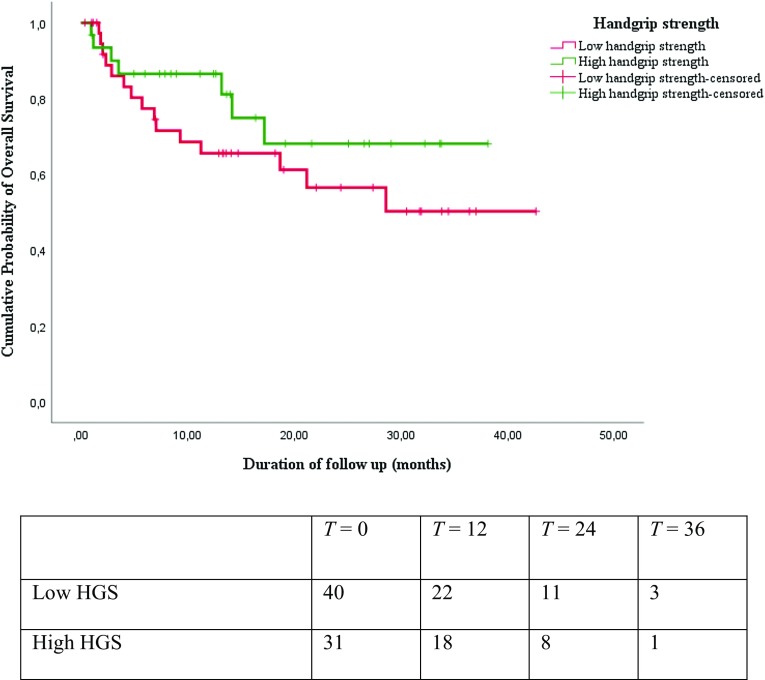
Kaplan–Meier overall survival curves and number at risk table for patients with low handgrip strength (HGS) and high HGS showed no statistically significant difference (Log-rank chi-square 1.35; *p* = 0.25)

**Fig. 3:**
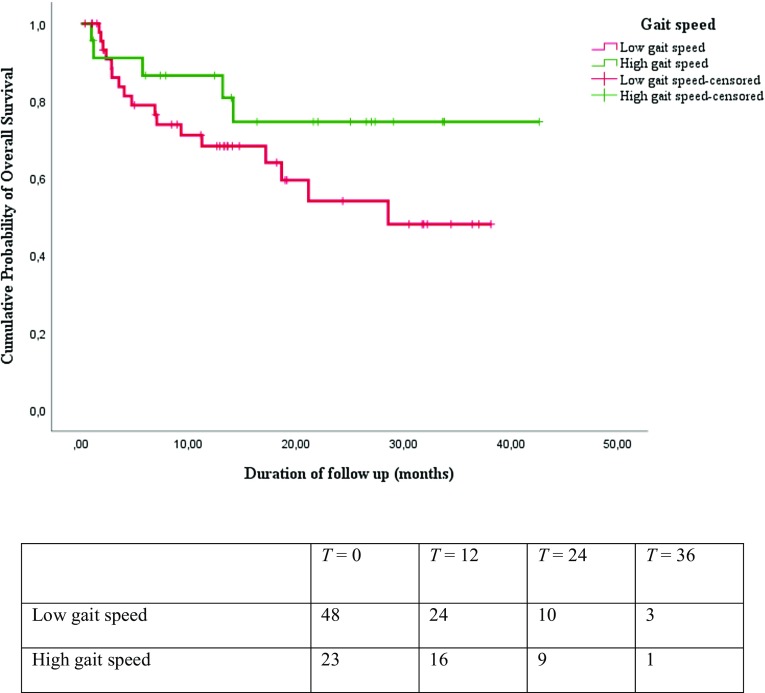
Kaplan–Meier overall survival curves and number at risk table for patients with low gait speed and high gait speed showed no statistically significant difference (Log-rank chi-square 1.95; *p* = 0.16)

**Fig. 4:**
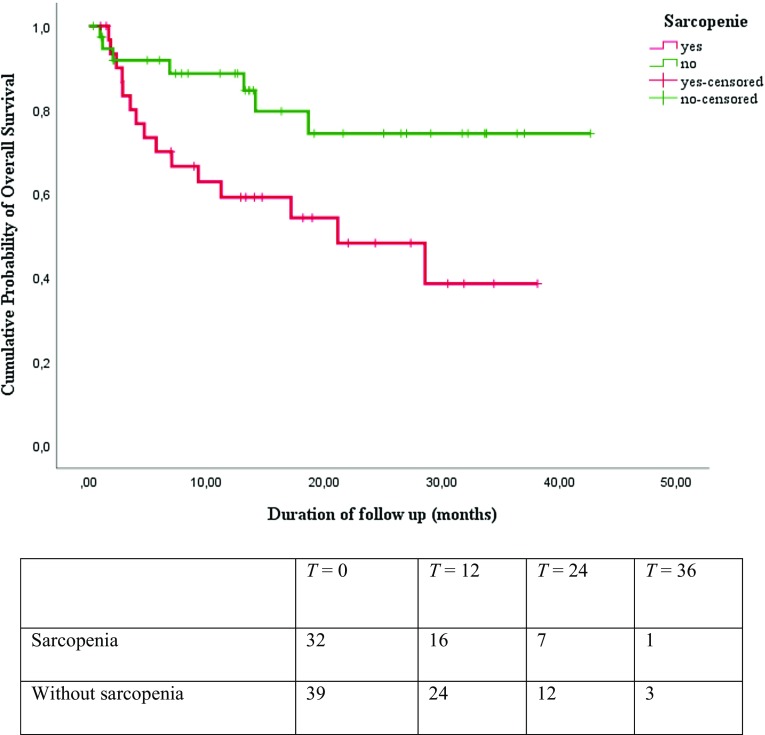
Kaplan–Meier overall survival curves and number at risk table for patients with and without sarcopenia showed statistically significant difference (Log-rank chi-square 5.50; *p* = 0.02)

Results from the Kaplan–Meier survival analysis are shown in Figs. 1, 2, 3, and 4. As shown in Figs. 1, 2, and 3, the median overall survival appears to be shorter for patients treated with curative intent with high SMI compared with patients with low SMI (10.58 versus 13.34 months; log-rank test *p* = 0.29), but this difference was not statistically significant. The differences in OS between patients with low HGS compared with patients with high HGS (13.31 versus 13.17 months; log-rank test *p* = 0.25) and for patients with low gait speed compared with patients with high gait speed (11.94 versus 16.36 months; log-rank test *p* = 0.16) were not significant either. The median overall survival was significantly shorter for patients treated with curative intent with sarcopenia compared to patients without sarcopenia (12.07 versus 13.60 months; log-rank test *p* = 0.02), as is illustrated in Fig. 4. The overall 3-year survival rate was significantly shorter for patients treated with curative intent with sarcopenia compared to patients without sarcopenia (39% versus 75%; Wilcoxon Statistic 4.48, *p* = 0.03).

Results from the univariate and multivariate cox regression analysis for overall survival are shown in Table 5. Sarcopenia (HR 2.80; 95% CI 1.14–6.88; *p* = 0.03) and TNM stage IV (HR 15.64; 95% CI 1.99–122.88; *p* = 0.01) were significant prognostic factors for overall survival in univariate cox regression analysis. In multivariate cox regression analysis, model 1 shows that sarcopenia (HR 2.66; 95% CI1.07–6.58; *p* = 0.04) remained a significant prognostic factor for overall survival independent of age, Hb level, BMI, MUST score, and comorbidity. However, sarcopenia did not remain a significant prognostic factor when TNM stage was included (model 2). TNM stage IV was a significant prognostic factor for overall survival in multivariate cox regression analysis (HR 15.64; 95% CI 1.99–122.88; *p* = 0.01).

**Table 5:** Univariate and multivariate analyses of the hazard ratios for sarcopenia, age, Hb level, BMI, MUST score, CCI, and TNM stage as independent prognostic factors for overall survival

Variable	Overall survival								
	Univariate analysis			Multivariate analysis(*)					
				Model 1			Model 2		
	HR	95% CI	*p* value	HR	95% CI	*p* value	HR	95% CI	*p* value
Sarcopenia	2.80	1.14–6.88	0.03*	2.66	1.07–6.58	0.04*	1.36	0.48–3.83	0.56
Age (years)	1.03	0.95–1.11	0.48	1.02	0.94–1.11	0.59	1.05	0.97–1.13	0.26
Hb (mmol/L)	0.82	0.58–1.16	0.26	0.90	0.61–1.32	0.58			
BMI (kg/m^2^)									
< 18.5	–	–	–	–	–	–	–	–	–
18.5–25	Ref			Ref			Ref		
25–30	0.54	0.21–1.38	0.20	0.54	0.21–1.39	0.20	0.50	0.19–1.31	0.16
≥ 30	0.34	0.10–1.20	0.10	0.45	0.12–1.64	0.23	0.70	0.18–2.72	0.61
MUST score									
< 2	Ref			Ref					
≥ 2	1.75	0.68–4.53	0.25	1.36	0.47–3.95	0.57			
CCI									
< 6	Ref			Ref			Ref		
≥ 6	1.22	0.52–2.86	0.65	0.92	0.35–2.40	0.86	1.47	0.58–3.74	0.42
TNM stage									
I	Ref			–	–	–	Ref		
II	0.96	0.06–15.40	0.98				0.96	0.06–15.40	0.98
III	5.24	0.57–48.46	0.14				5.24	0.57–48.46	0.14
IV	15.64	1.99–122.88	0.01*				15.64	1.99–122.88	0.01*

*Correlation is significant at the 0.05 level (two-tailed)

(*)Model 1 includes the variables; sarcopenia, age, hb value, BMI, MUST score, and CCI. Model 2 includes the variables; sarcopenia, age, BMI, CCI, and TNM stage

A subgroup analysis according to TNM stage was performed, of which the results are shown in Table 6. Sarcopenia was a statistically significant prognostic factor for overall survival in patients with TNM stage I–III (HR 9.19; 95% CI 1.07–78.74; *p* = 0.04). However, sarcopenia was not a statistically significant prognostic factor for overall survival in patients with TNM stage IV (HR 0.90; 95% 0.32–2.55; *p* = 0.85).

**Table 6:** Subgroup analyses according to TNM stage and sarcopenia showed sarcopenia as a statistically significant prognostic factor for overall survival in all patients with curative treatment intention (HR 2.80; 95% CI 1.14–6.88; *p* = 0.03) and in all patients with TNM stage I–III (HR 9.19; 95% CI 1.07–78.74; *p* = 0.04)

Subgroup	Overall survival			
	Sarcopenia			
	Frequency	HR	95% CI	*p* value
TNM stage I–III	32	9.19	1.07–78.74	0.04*
TNM stage IV	39	0.90	0.32–2.55	0.85
Curative treatment intention	71	2.80	1.14–6.88	0.03*

*Correlation is significant at the 0.05 level (two-tailed)

## Discussion

Sarcopenia is a common and highly prevalent clinical problem in the elderly patient. Literature showed that sarcopenia is associated with several negative outcomes; however, literature mainly focuses on radiologically assessed low SMM rather than the combination of SMM and MF [6–16]. In addition, no studies report on the impact of sarcopenia on survival in the elderly head-and-neck cancer patient. Identification of the impact of low SMM and low MF on prognosis in the elderly head-and-neck cancer patient will stimulate the development of novel interventions to gain SMM and MF which may improve the prognosis of these patients. Regardless of the success of an intervention, information on prognosis can be used for patient counseling and treatment decision making.

In this study, we included 85 patients of whom 41 patients (48.2%) were classified as sarcopenic. This number is in accordance with recent medical literature which estimated the prevalence of sarcopenia in elderly patients diagnosed with different types of cancer between 14 and 78.7% [22]. The prevalence estimates of sarcopenia in the elderly non-cancer patients are lower, ranging between 5 and 50%. Sarcopenia is prevailing in elderly cancer patients because of the frequent weight loss caused by low food intake, increased catabolic pathways, increased inflammation, increased lipolysis, and increased proteolysis associated with both old age and malignancy [22].

This study shows that SMM, muscle strength, and physical functioning separately had no significant prognostic value for overall survival. A combination of muscle mass and muscle strength or muscle performance did show a significant prognostic value for overall survival in elderly patients with head-and-neck cancer. This is in accordance with previous studies in other tumor types, which have demonstrated that not only SMM but also MF is related with several health outcomes [22–24]. Previous studies in patients with esophageal cancer did not show a significant prognostic value of sarcopenia on overall survival; however, sarcopenia was defined as low radiologically assessed SMM only rather than a combination of low SMM and low MF [25–28]. Our study highlights the importance of defining sarcopenia as a combination of SMM and MF.

In multivariate analysis including the covariates age, Hb level, MUST score, BMI, and comorbidity; sarcopenia remained a statistically significant prognostic factor for overall survival. When including TNM stage in the multivariate analysis, sarcopenia did not remain a statistically significant prognostic factor for overall survival. Subgroup analyses according to TNM stage and treatment intention shows that sarcopenia is a statistically significant prognostic factor for overall survival in patients with TNM stage I–III and in all patients with curative treatment intention. In patients with TNM stage IV, sarcopenia is not a statistically significant prognostic factor for overall survival. In this study, 39 patients (45.9%) had a TNM stage IV; it is possible that sarcopenia did not remain a significant prognostic factor in model 2 of the multivariate analysis because of the high number of patients with TNM stage IV. This finding is in accordance with a previous study performed in patients with gastric cancer which showed that sarcopenia is a significant prognostic factor for overall survival in patients with TNM stage II–III [30]. It is also in accordance with a recent systematic review, which showed that sarcopenia is a significant prognostic factor for overall survival in different types of cancers independent of TNM stage [29].

The existing literature on sarcopenia in patients with head-and-neck cancer is scarce and focuses mainly on low SMM in patients who receive (chemo)radiotherapy [7] or patients who undergo a total laryngectomy [10, 11]. To our knowledge, our study is the first to investigate the impact of sarcopenia, defined as a combination of SMM and MF, in elderly (≥ 70-years old) head-and-neck cancer patients.

This study has some limitations. It was designed as a retrospective single-center study, which increases the risk for systemic errors. It had limited number of included patients which may have led to type II errors. Only patients with the available data on SMM and MF were included in the study. As it is more likely that MF parameters were examined for frail patients than for fit patients, this may have resulted in a biased study population in which it is probably more difficult to show the prognostic value of sarcopenia. Therefore, sarcopenia a combination of SMM and MF should be further evaluated as a prognostic factor for overall survival in elderly patients with head-and-neck cancer.

Concerning the imaging techniques used to assess SMM, we decided to include both CT scans and MRI scans of the head and neck area to assess SMM, to maximize the number of patients that could be included. Whenever available, we used CT imaging instead of MRI, because most research on SMM in cancer patients is performed using CT imaging. However, the CT measurement method for SMM was formulated on MRI-based research [30, 31]. Theoretically, there is no difference in SMM between CT imaging and MRI, as both methods are very accurate for SMM assessment. Therefore, we believe that it is acceptable to use MRI for the SMM measurement when CT imaging is not available. Research should be conducted to investigate this further.

In retrospective studies, data on MF will probably rarely be available, whereas CT or MRI is often routinely performed in head-and-neck cancer patients. We propose to conduct further prospective studies for the measurement of both MF and SMM and to perform routine handgrip strength measurements in every newly diagnosed head-and-neck cancer patient.

In conclusion, sarcopenia is present in half of the elderly HNSCC patients. Skeletal muscle mass index and muscle function, as determined by muscle strength or physical performance measurements, were not prognostic separately in elderly HNSCC patients, but the combination of both was prognostic for overall survival. Therefore, it may be preferable to define sarcopenia as the combination of low skeletal muscle mass and low muscle function and not by radiologically assessed skeletal muscle mass alone.

## Abbreviations

SMM: Skeletal muscle mass
HNC: Head-and-neck cancer
C3: Third cervical vertebra
L3: Third lumbar vertebra
CSMA: Cross-sectional muscle area
HU: Hounsfield unit
Lumbar SMI: Lumbar skeletal muscle index
